# Trends in Nutrition Research for Sarcopenia: A Bibliometric Analysis

**DOI:** 10.3390/nu14204262

**Published:** 2022-10-12

**Authors:** Lei Wu, Kelin He, Dandan Fang, Xiuyue Qiu, Wenhui Xiao, Shuhui Lou, Rulin Yong

**Affiliations:** 1 Department of Acupuncture, The Third Affiliated Hospital of Zhejiang Chinese Medical University, Hangzhou 310005, China; 2 Department of Neurobiology and Acupuncture Research, The Third School of Clinical Medicine (School of Rehabilitation Medicine), Zhejiang Chinese Medical University, Key Laboratory of Acupuncture and Neurology of Zhejiang Province, Hangzhou 310053, China; 3 The Third School of Clinical Medicine (School of Rehabilitation Medicine), Zhejiang Chinese Medical University, Hangzhou 310053, China; 4 School of Nursing, Zhejiang Chinese Medical University, Hangzhou 310053, China

**Keywords:** sarcopenia, nutrition, older people, bibliometrics, quantitative analyses

## Abstract

Sarcopenia is age-related, pathophysiological muscle atrophy. Research regarding nutrition treatment of sarcopenia has developed rapidly, particularly as populations age. We evaluated the development of trends in this field using a bibliometric analysis. Articles up to July 2022 were searched in the Scopus database. Bibliographic information from the selected publications, such as countries, citations, world maps, institutions, authors, journals, and keywords, were converted and analyzed automatically using the “bibliometrix” package in R environment (version 4.2.0). We identified 368 Scopus articles from 1998–2021. According to citation analyses, 27 documents received more than 100 citations. Bibliometric analysis based on the literature included in this study revealed that South Korea (61 publications), United States (50), Japan (35), China (30), and Italy (20) contributed the most publications. Tehran University of Medical Science (19 records, 2.55% of articles) contributed the most publications. The most productive author was Landi, with eight articles (2.17% of articles). The publications were located in 196 journals, with *Nutrients* having the most publications (30, 8.15% of articles). The curves representing keywords “sarcopenia” and “aged” were the most apparent. Our analysis suggested that global nutrition and sarcopenia-related research increased rapidly from 2001 to 2021, demonstrating that this was a promising area of future research that could benefit from continued advances. Future research may focus on the effects of age and sex as well as intervention effectiveness, particularly exercise and nutrition supplementation.

## 1. Introduction

Sarcopenia is age-related, pathophysiological muscle atrophy characterized by muscle loss, weakened muscle strength, and declining physical performance [1]. According to estimates, the population of older adults (defined as those aged > 60 years) will double by the year 2050, from 841 million to 2 billion, or 21% of the global population [2]. Sarcopenia is common in older adults, with an estimated prevalence of 5–13% in adults aged 60–70 years and 11–50% in adults aged > 80 years [3]. Sarcopenia in older people is a serious problem for society [4], making the improvement in preventive and therapeutic strategies vital. Moreover, the World Health Organization Health Report has noted the issue of sarcopenia, and musculoskeletal education has been recognized as a national and global priority.

Malnutrition plays a major role in the pathogenesis of sarcopenia. The benefits of proper nutrition include reduced loss of muscle mass and function, as well as prolonged independence and quality of life [5]. Managing the diet of sarcopenic older patients should begin at diagnosis, with assessments of their nutritional status and the provision of nutritional counselling. The effects of energy and protein intake as well as other key nutrients on muscle may be a popular direction for future research [6].

Bibliometric analysis as a research method is the application of quantitative methods in representing scientific literature visually [7]. Recently, it has been widely used to analyze published literature in different fields, such as sarcopenia research [8,9,10,11,12]. In the past decade, research regarding nutrition in the treatment of sarcopenia has developed rapidly, with several highly regarded publications being published [13,14,15,16,17]. Therefore, carrying out bibliometric research related to nutrition and sarcopenia may have reference significance for the next stage of development in this field. A bibliometric analysis which reviews all sarcopenia-related research trends over the previous 20 years and exclusively emphasize the value of nutrition in the conclusion [6]. However, it is not enough to reflect the research trends in nutrition research for sarcopenia, as that the role of it has always attracted great attention. So, in this study, we mainly focus on using the “bibliometrix” package in R environment (version 4.2.0) to analyze the research status of nutrition in sarcopenia, and the future research in this field will be guided by the results of this study. 

## 2. Materials and Methods

### 2.1. Data Sources and Search Strategies

Data were obtained from the Web of Science Core Collection (WOS) and Scopus databases. Based on previous study protocols [18], keywords used for participant conditions were “sarcopenia” OR “frailty/frail” OR “older/elderly”. Keywords used for interventions were “diet” OR “diets” OR “dietary” OR “nutrition” OR “supplementation” OR “protein”. There were several types of articles that had search terms in their titles, and all queries had been completed as of 2 July 2022. 

### 2.2. Data Collection and Bibliometric Analysis

**Figure 1:**
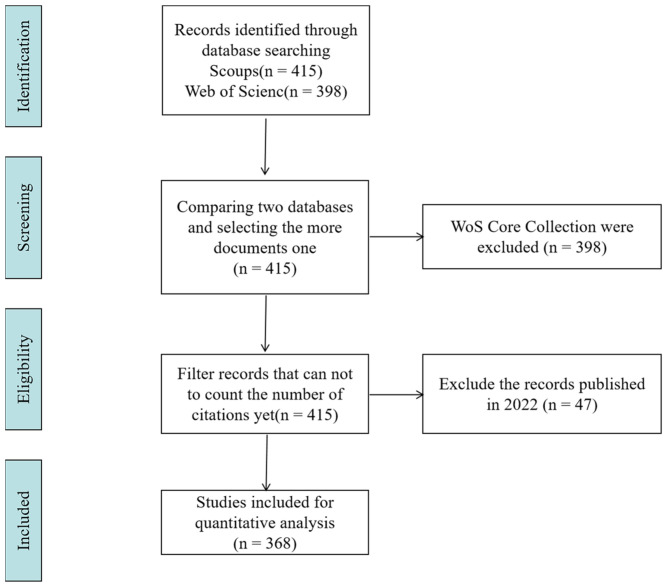
Flow diagram of study selection procedure.

As of 2 July 2022, 813 records were noted. Of them, 415 were from Scopus and 398 were from WOS. Scientific literature can be found in both WOS and Scopus, the two most important databases. However, for citation analyses, Scopus offered approximately 20% more coverage than WOS [19]. Therefore, we used the Scopus database solely for bibliometric analysis. After filtering reports published in 2022, which may not yet include the number of citations, 368 studies were included in the quantitative analysis. An overview of the selection process for studies is provided using the Preferred Reporting Items for Systematic Reviews and Meta-Analyses (PRISMA) flow diagram in Figure 1.

In R (R Project for Statistical Computing, http://www.r-project.org/ (accessed on 18 June 2022), R version 4.2.0), the bibliographic information of the selected publications was automatically converted and analyzed using the “bibliometrix” function [20,21]. We analyzed all information related to sources, countries, citations, world map, institutions, authors, journals, and keywords.

## 3. Results

### 3.1. Data Descriptive Analysis

Briefly, 368 documents were found to have 31.67 average citations. Ten document types were included, such as articles, books, book chapters, conference papers, editorials, errata, letters, notes, reviews, and short surveys. A total of 2017 authors relevant to the topic were identified in this search, among whom, 36 were single authors and 1981 were part of multi-authored studies, indicating that most of the publications were co-authored. The average number of co-authors per document was 6.67, and the international co-authorship percentage was 16.58%.

### 3.2. Global Trends in the Publications

**Figure 2:**
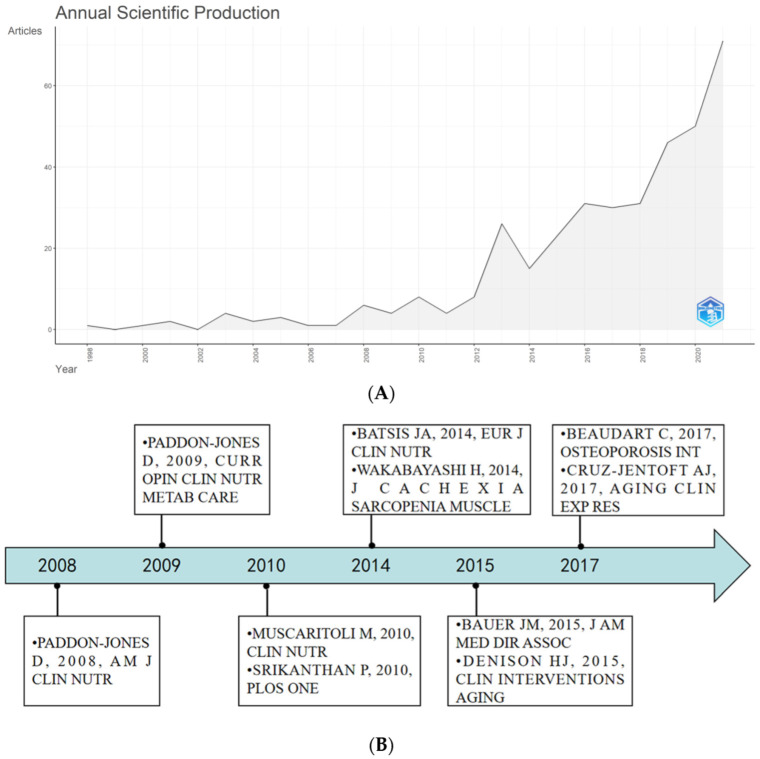
(**A**) The number of nutrition research works on sarcopenia. (**B**) Timeline of the top 10 cited documents.

A total of 368 articles from 1998–2021 related to nutrition and sarcopenia were retrieved from Scopus. From one article (0.27%) in 1998 to 71 articles (19.29%) in 2021, global trends have increased steadily since 1998. The annual growth rate was 20.36% (Figure 2A).

### 3.3. Analysis of Citations

The citation analysis showed that 27 documents had over 100 citations. Figure 2B shows the top 10 documents that had the most citations, including 1025 citations in “Consensus definition of sarcopenia, cachexia and pre-cachexia: Joint document elaborated by Special Interest Groups (SIG) ‘cachexia-anorexia in chronic wasting diseases’ and ‘nutrition in geriatrics’” (Muscaritoli et al., 2010); 555 citations in “Dietary protein recommendations and the prevention of sarcopenia” (Paddon-Jones et al., 2009); and 373 citations in “Effects of a Vitamin D and Leucine-Enriched Whey Protein Nutritional Supplement on Measures of Sarcopenia in Older Adults, the PROVIDE Study: A Randomized, Double-Blind, Placebo-Controlled Trial” (Bauer et al., 2015).

### 3.4. Analysis of Countries

**Figure 3:**
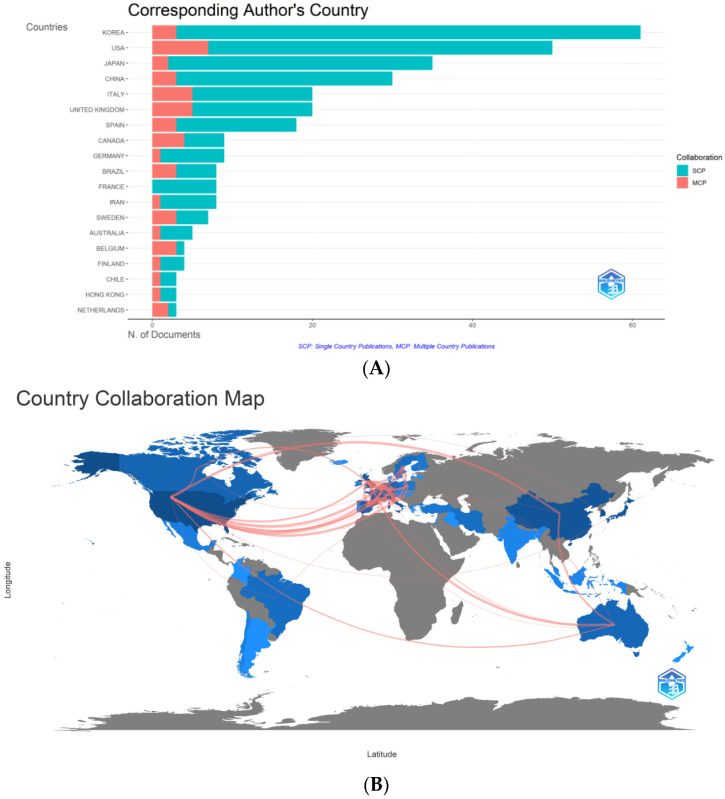
(**A**) Most productive countries and regions in the field of nutrition research works on sarcopenia. (**B**) The collaboration between countries and regions on nutrition research related to sarcopenia. The colour segmentation includes blue (with publications) and grey (without publications). The thickness of the red lines indicates the number of co-published papers. The colour intensity corresponds to the number of publications).

Based on the countries of the corresponding authors, the top five countries with the most publications were South Korea, the United States, Japan, China, and Italy. South Korea contributed the most articles (61, 16.58%), followed by the United States (50, 13.59%), Japan (35, 9.51%), China (30, 8.15%), and Italy (20, 5.43%) (Figure 3A). Furthermore, the collaboration map in this field was analyzed for each country or region (Figure 3B). The United Kingdom and Italy had the most collaborations, with 12 papers in collaboration.

### 3.5. An Analysis of Institutions

**Figure 4:**
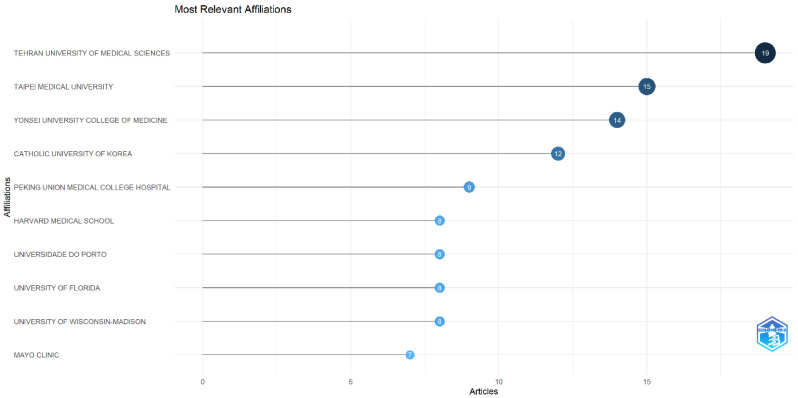
Top 10 institutions performing nutrition research works on sarcopenia.

Figure 4 shows the institutions involved in the research, with a total of 744 institutions. Nineteen publications were contributed by Tehran University of Medical Science (2.55%), followed by Taipei Medical University (15, 2.02%), Yonsei University College of Medicine (14, 1.89%), Catholic University of Korea (12, 1.61%), and Peking Union Medical College Hospital (9, 1.21%).

### 3.6. An Analysis of the Authors

**Figure 5:**
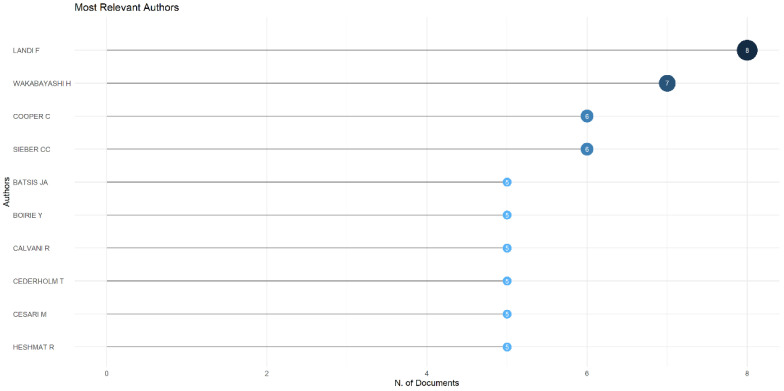
Top 10 authors that produced nutrition research works on sarcopenia.

A summary of the top 10 most productive authors is shown in Figure 5. Landi produced the most publications, with eight articles (2.17%), followed by Wakabayashi with seven articles (1.90%), Cooper (6, 1.63%), Sieber (6, 1.63%), and Batsis (5, 1.36%).

### 3.7. Analysis of Journals

**Figure 6:**
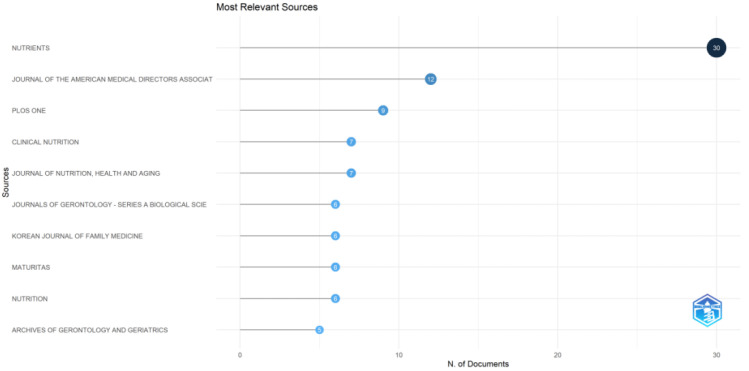
Top 10 journals publishing nutrition studies on sarcopenia.

The 368 analyzed articles were published in 196 journals, and Figure 6 shows the top 10 most popular journals. The largest number of publications came from *Nutrients* (30, 8.15%), followed by *Journal of the American Medical Directors Association* (12, 3.26%), *PLOS ONE* (9, 2.45%), *Clinical Nutrition* (7, 1.90%), and *Journal of Nutrition* (7, 1.90%).

### 3.8. Analysis of Keywords

**Figure 7:**
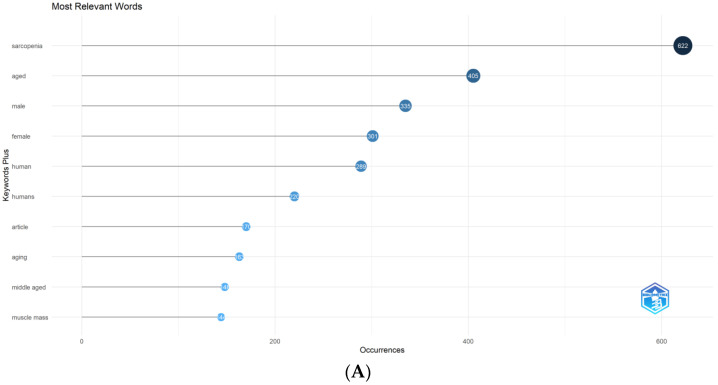

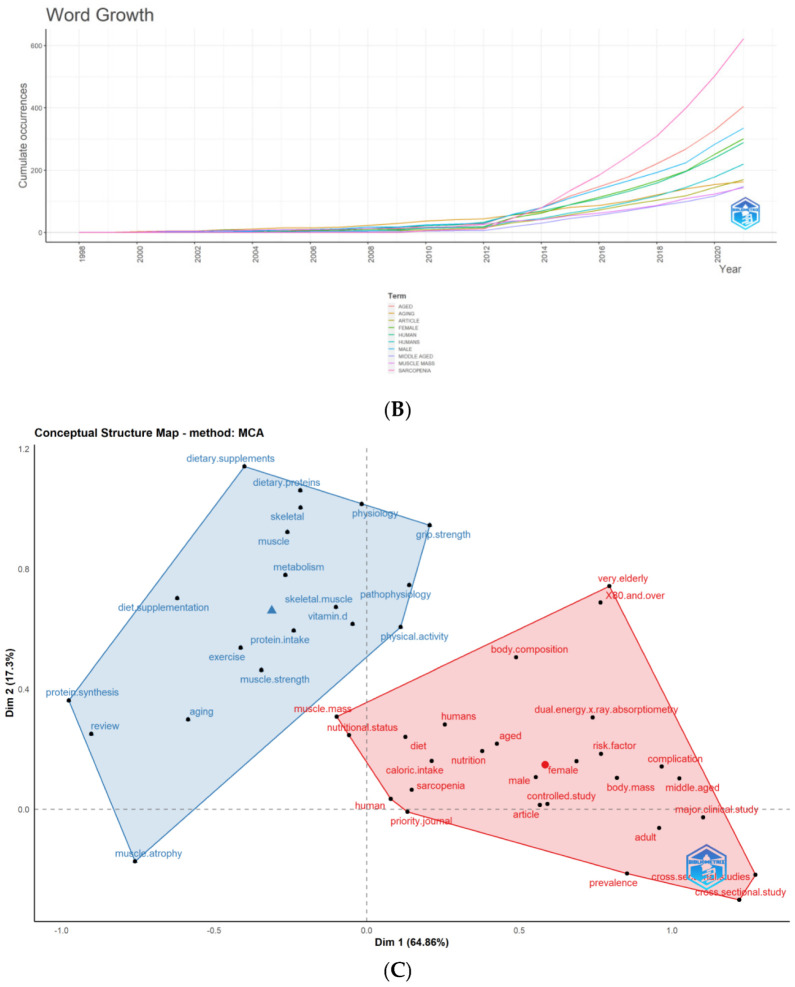
(**A**) The top 10 most relevant keywords in nutrition research for sarcopenia. (**B**) The top 10 most apparent trends in nutrition research for sarcopenia. (**C**) Nutrition research for sarcopenia conceptual structure map (map dimensions: average position of publications; map midpoint: centre of research area).

The total number of keywords occurring more than five times was 425. Figure 7A shows the most relevant keywords, while Figure 7B shows the most obvious trends: two curves corresponding to sarcopenia and aging separately. Based on the multidimensional scaling method, the clustered topics could be divided into two categories [22], as shown in Figure 7C. There are two main clusters in the conceptual structure map of nutrition research for sarcopenia. According to one cluster, sarcopenia was associated with differences in age and sex, while the other examined the effectiveness of exercise and nutrition supplementation in sarcopenia.

## 4. Discussion

Population aging has become an important social issue worldwide [23]. Globally, the population of people aged > 70 years has increased from 1990–2019. In particular, there has been an increase of 115.4% in the population aged 70–79 years, 164.7% in those aged 80–94 years, and 363.7% in those aged ≥ 95 years. Compared with 1990, the population aged 70–79 years increased by 168.3 million individuals in 2019. Additionally, those aged 80–94 increased by 90.1 million, and those aged ≥ 95 years increased by 3.7 million [24]. According to the World Population Prospects 2019, 1 in 6 people in the world are expected to be over 65 years of age (16%) by 2050, up from 1 in 11 in 2019 (9%). Over the next 50 years, the share of people aged ≥ 65 is projected to double in Northern Africa, Western Asia, Central Asia, Southern Asia, Eastern and Southeast Asia, Latin America, and the Caribbean. Moreover, the number of people aged > 65 in Europe and Northern America could rise to one in four by 2050. Globally, individuals over 65 years of age outnumbered children under five for the first time ever in 2018. Moreover, by 2050, there will be 426 million older people, up from 143 million in 2019. Increasing geriatric populations lead to a rise in sarcopenia, a geriatric syndrome characterized by progressive loss of skeletal muscle mass and function [25]. Developing a nomenclature for aging-related skeletal muscle degeneration was a major step towards reaching international consensus on the issue in 2016 [26]. We can also see from the figure that the literature on this disease has increased yearly since 2016. 

In this study, we evaluated the publishing trends related to nutritional interventions in the research of sarcopenia and closely examined the countries, journals, and authors that contributed to this emerging field. From one article in 1998 to 71 articles in 2021, the global trends have increased steadily since 1998. Based on the country of the corresponding author, the five countries with the most publications were South Korea, the United States, Japan, China, and Italy. Apart from China, the main countries engaged in this research were all developed countries with an aging population, especially Japan and Italy. According to provisional estimates by governments, in 2015, 26.7%, 14%, and 22% of the populations of Japan, the United States, and Italy, respectively, were aged > 65 [27]. Additionally, as of 2050, older people will comprise 35.9% of the South Korean population [28]. According to The World Population Prospects 2019, by 2035, those in China aged ≥ 60 will reach 410 million, accounting for 28.4% of the population. This estimate is 10.6 percentage points higher than the overall level of the world and 2.1 percentage points lower than that of developed countries. China is on the cusp of a heavily aging society and has introduced many policies, including promoting the research and development of the standardized management of nutritional food for older populations [29]. In addition, members of various working groups on sarcopenia have promoted the number of papers published in their countries, including experts from Italy in the European Working Group on Sarcopenia in Older People (EWGSOP2) [30]. Landi, from Università Cattolica del Sacro Cuore and a member of the EWGSOP2, was the most productive author according to our results. Additionally, among the 23 authors of the article published by the Asian Working Group for Sarcopenia in 2019, 6 (26.9%) were from South Korea and 6 (26.9%) were from Japan [31]. Our results also indicated that the largest number of publications came from the journal *Nutrients* as well as the Tehran University of Medical Science institution. These various results may indicate potential future directions of cooperative research and publishing. 

Clustered topics could be divided into two main clusters in the conceptual structure map of nutrition research in sarcopenia by using the multidimensional scaling method. One cluster involved the risk factors of sarcopenia, including sex and age, while the other represented the interventions for sarcopenia, such as exercise and nutrition. To improve health, nutrition guidelines should be consistent. In China, one of the factors that contributed most to disability-adjusted life years (DALYs) is dietary factors [32]. Nutritional disorders, including dietary iron deficiency and protein-energy malnutrition, are highlighted in the GBD 2019 Diseases and Injuries report, and in terms of absolute declines in DALYs between 1990 and 2019, these disorders were among the 10 largest contributors [33]. 

Malnutrition is one of the causes of sarcopenia, and supplementing protein and amino acids may increase muscle protein synthesis and improve symptoms. According to existing research, older people with and without sarcopenia consume different amounts of calories, macronutrients, such as protein, and micronutrients, such as calcium and vitamins [34]. A systematic review and meta-analysis by Yoshimura et al. indicated that after three months of nutritional intervention, muscle strength improved [35]. Moreover, a systematic review and meta-analysis by Hanach et al. found that there were significant increases in appendicular muscle mass after consuming dairy protein [36]. An umbrella review by Gielen et al. also found that sarcopenia sufferers benefitted significantly from leucine’s muscle-building properties [37]. The results of a scoping systematic review and meta-analysis by Bird et al. indicated that body muscle mass and strength were positively affected by Omega-3 long chain polyunsaturated fatty acids (LC PUFAs) supplementation [38]. A network meta-analysis by Cheng et al. showed that vitamin D supplementation may be helpful in regaining function in people with sarcopenia [39]. Additionally, a systematic review and network meta-analysis by Wu et al. found that exercise and nutrition may improve physical performance in older adults with sarcopenia by increasing muscle strength and reducing muscle loss [40], and a network meta-analysis by Negm et al. showed that the most effective sarcopenia treatment was a combination of exercise, physical activity, and nutritional supplementation [41]. Finally, a meta-analysis by Huang et al. examined how omega-3 fatty acids affect muscle mass, particularly when taken at levels higher than 2 g per day. Additionally, long-term supplementation with omega-3 fatty acids may improve walking speed [42].

Overall, the role of nutrition in sarcopenia has always attracted great attention. Healthcare professionals are encouraged by the ESWGOP to seek answers to questions such as “what amounts of macronutrients and micronutrients are needed for older people with sarcopenia?”, “Is there a difference in how meals are taken and/or when supplements are taken [43]?” and “what is the pathogenesis of sarcopenia?”. These questions may require further study in the future.

## 5. Conclusions

Our analysis suggested that global nutrition and sarcopenia-related research has increased rapidly, with South Korea being the leader in terms of countries. Additionally, the Università Cattolica del Sacro Cuore’s Francesco Landi is one of the world’s leading scientists in this field. Future research may continue to focus on the differences in age and sex, as well as the effectiveness of interventions, including exercise and nutrition supplementation. Researchers can design future studies by understanding the leading authors, journals, institutions, and citations. They will further be able to decide the direction of their research based on the emerging trends. However, it is important for researchers to be aware of the limitations when using databases for bibliometric analysis, not all journals in every discipline are covered by Scopus. Furthermore, non-English language journals are underrepresented.

## Data Availability

The raw data used in this article can be obtained from the Scopus and Web of Science (WoS) Core Collection database, further inquiries can be directed to the corresponding author.
